# Calmodulin kinase II regulates amphetamine-induced reverse transport in dopamine and serotonin transporters

**DOI:** 10.1186/2050-6511-13-S1-A56

**Published:** 2012-09-17

**Authors:** Thomas Steinkellner, Therese Montgomery, Jae-Won Yang, Matthias Rickhag, Sonja Sucic, Ype Elgersma, Oliver Kudlacek, Michael Freissmuth, Ulrik Gether, Harald H Sitte

**Affiliations:** 1 Institute of Pharmacology, Center for Physiology and Pharmacology, Medical University Vienna, 1090 Vienna, Austria; 2 School of Biomolecular and Biomedical Science, University College Dublin, Ireland; 3 Molecular Neuropharmacology Group and Center for Pharmacogenomics, Department of Pharmacology, The Panum Institute, University of Copenhagen, 2200 Copenhagen, Denmark; 4 Department of Neuroscience, Erasmus University Medical Center, 3015 GE Rotterdam, The Netherlands

## Background

Monoamine transporters such as the dopamine transporter (DAT) and the serotonin transporter (SERT) mediate the reuptake of previously released monoamines dopamine (DA) and serotonin from the synaptic cleft; thereby, these transporters regulate the monoamine content available for synaptic transmission. Certain stimuli, such as changes in ionic composition of the extracellular fluid or psychostimulants (e.g. amphetamines) are able to induce outward transport and thus increase extracellular monoamine concentrations. Influx and efflux of substrate are thought to be asymmetrical processes regulated by intracellular kinases. It has been demonstrated that removal of N-terminal serines ablates amphetamine-induced reverse transport in the DAT. Furthermore, the Ca^2+^/calmodulin-dependent protein kinase II a (aCaMKII) can bind to the DAT C-terminus and phosphorylate N-terminal serines. Pharmacological inhibition of aCaMKII dramatically reduces amphetamine-induced efflux both in cells stably transfected with the human DAT as well as in rat striatal slices. Here, we test whether aCaMKII-regulation of amphetamine-induced reverse transport of monoamines is affected in mice with mutations in the aCaMKII gene.

## Methods

Methods used were: release assays in mouse brain preparations, radioligand binding and uptake experiments, immunoprecipitations, surface biotinylation, mass spectrometry, primary cultures of dopaminergic and serotonergic neurons, immunocytochemistry and behavioural pharmacology.

## Results

We show here that aCaMKII regulates amphetamine-induced DAT-mediated efflux in mice with various mutations in the aCaMKII gene. Mice lacking aCaMKII or having a permanently self-inhibited aCaMKII (aCaMKII^T305D^) display significantly reduced amphetamine-induced substrate efflux. A similar finding was observed in a mouse model of Angelman Syndrome, a neurogenetic disease characterized by motor impairments and autism spectrum disorders. Angelman Syndrome mice have a reduced aCaMKII activity and show comparable impairments in DAT function to aCaMKII mutants. This suggests that DAT-mediated dopaminergic signalling is affected in Angelman Syndrome. We further show that aCaMKII regulates the closely related SERT: both pharmacological inhibition and genetic disruption of aCaMKII significantly attenuates *p*-chloro-amphetamine-induced SERT-mediated serotonin efflux in transiently transfected cells and mouse brain preparations.

## Conclusions

aCaMKII exerts an important modulatory role in amphetamine-induced DAT- and SERT-mediated substrate efflux. The finding that efflux is also affected in Angelman Syndrome mice might help in the understanding of the underlying pathophysiology. Symptoms of human Angelman Syndrome patients include movement impairments and autism spectrum disorders, conditions which are associated with dopaminergic and serotonergic malfunction.

